# Development of Low-Cost Air Quality Stations for Next Generation Monitoring Networks: Calibration and Validation of PM_2.5_ and PM_10_ Sensors

**DOI:** 10.3390/s18092843

**Published:** 2018-08-28

**Authors:** Alice Cavaliere, Federico Carotenuto, Filippo Di Gennaro, Beniamino Gioli, Giovanni Gualtieri, Francesca Martelli, Alessandro Matese, Piero Toscano, Carolina Vagnoli, Alessandro Zaldei

**Affiliations:** 1 Department of Information Engineering (DINFO), University of Firenze, Via di Santa Marta 3, 50139 Firenze, Italy; alice.cavaliere@unifi.it; 2 National Research Council-Institute of Biometeorology (CNR-IBIMET), Via Caproni 8, 50145 Firenze, Italy; f.carotenuto@ibimet.cnr.it (F.C.); f.digennaro@ibimet.cnr.it (F.D.G.); beniamino.gioli@cnr.it (B.G.); f.martelli@ibimet.cnr.it (F.M.); a.matese@ibimet.cnr.it (A.M.); p.toscano@ibimet.cnr.it (P.T.); c.vagnoli@ibimet.cnr.it (C.V.); a.zaldei@ibimet.cnr.it (A.Z.)

**Keywords:** air quality monitoring, low-cost sensors, next generation networks, laboratory calibration, field validation, PM_2.5_, PM_10_

## Abstract

A low-cost air quality station has been developed for real-time monitoring of main atmospheric pollutants. Sensors for CO, CO_2_, NO_2_, O_3_, VOC, PM_2.5_ and PM_10_ were integrated on an Arduino Shield compatible board. As concerns PM_2.5_ and PM_10_ sensors, the station underwent a laboratory calibration and later a field validation. Laboratory calibration has been carried out at the headquarters of CNR-IBIMET in Florence (Italy) against a TSI DustTrak reference instrument. A MATLAB procedure, implementing advanced mathematical techniques to detect possible complex non-linear relationships between sensor signals and reference data, has been developed and implemented to accomplish the laboratory calibration. Field validation has been performed across a full “heating season” (1 November 2016 to 15 April 2017) by co-locating the station at a road site in Florence where an official fixed air quality station was in operation. Both calibration and validation processes returned fine scores, in most cases better than those achieved for similar systems in the literature. During field validation, in particular, for PM_2.5_ and PM_10_ mean biases of 0.036 and 0.598 µg/m^3^, RMSE of 4.056 and 6.084 µg/m^3^, and R^2^ of 0.909 and 0.957 were achieved, respectively. Robustness of the developed station, seamless deployed through a five and a half month outdoor campaign without registering sensor failures or drifts, is a further key point.

## 1. Introduction

Air quality has a huge impact on the quality of life, and long-term exposure to polluted air can result in permanent health issues [1]. A number of epidemiological studies have clearly linked atmospheric pollutants to asthma, bronchitis, heart attacks and strokes [2,3,4]. For these reasons, air quality monitoring is required by national air quality regulations, such as the European Directive 2008/50/EC [5]. The equipment necessary to meet the standards established by these regulations on air quality monitoring has a high cost of procurement and maintenance [1]. Monitoring of air pollutants is primarily performed using analytical instruments, such as optical and chemical analysers [6]. Usually, air pollutant analysers are complicated, bulky and expensive, with each instrument costing anywhere from about 6000 to tens of thousands euros, together with a significant amount of resources required to routinely maintain and calibrate them [7,8]. Setting up and managing networks of fixed air quality stations also require significant investment [6]. Spread sparsely around or within a particular city, these stations can provide detailed and highly accurate measurements [8], but at limited point-based locations. This makes it difficult to compile representative and reliable information for a whole urban area, and thereby, to form a clearer picture of pollution field trends [6].

Alternatives to existing high-cost sparse fixed monitoring stations have been discussed and investigated previously by several groups (e.g., [9,10]). Recently, there has been an emerging trend of using the next generation air sensors to provide highly resolved air quality data as an alternative or integrative monitoring option. Miniaturization and other technological advances have brought to market a number of low-cost (<€2000) sensors designed to measure atmospheric particles and gases [11,12,13], including for example: CO [14,15,16], NO_2_ [15,16,17,18], O_3_ [1,15,16,17,19,20], VOC [21,22], PM_2.5_ [16,22,23,24,25,26,27], PM_10_ [1,25], and black carbon [28]. Due to their low cost, small size and low power consumption, the new technologies are very appealing in situations where traditional monitors are impractical [6,29]. Aside from supplying a more widespread network able to effectively capture the spatio-temporal variability of air pollution, the deployment of low-cost sensors in significant numbers can also assist in detecting pollution hotspots, assessing real-time exposure for designing mitigation strategies, and creating pollutant emission inventories once local atmospheric conditions and sources (e.g., cars) operational conditions are known [6]. In any case, these low-cost devices are not meant to replace official air control systems, but to integrate them [11,30]. The 2008/50/EC EU Directive [5], currently in force in Europe, has in fact introduced the concept of “indicative measurements”, intended as measurements which shall meet data quality objectives less strict than those required for fixed measurements. To provide adequate information on air quality spatial distribution, particularly within zones or agglomerations where upper pollution thresholds are exceeded, this directive states that indicative measurements may be used for supplementing air pollution levels measured by fixed monitoring stations. The legislative importance of indicative measurements is therefore to be stressed, as “the results of […] indicative measurement shall be taken into account for the assessment of air quality with respect to the limit values” [5,7].

Nevertheless, some drawbacks of low-cost sensors should be borne in mind. These include, aside from lower sensing accuracy than traditional monitoring stations [11]: (i) sensitivity to environmental factors like temperature, humidity, and pressure; (ii) degradation of sensing accuracy with age; (iii) sensor drifts, requiring frequent recalibration; (iv) slowness to respond to changes in pollutant levels, causing pollutant bursts encountered in mobile deployments to be underestimated.

To pursue the goal of providing air quality indicative measurements, a low-cost air quality station—named “AIRQino”—has been developed for real-time monitoring of main (both gaseous and particulate) atmospheric pollutants. The station has been undergone to a laboratory calibration, and later to a field validation. At this first stage, both calibration and validation processes were applied to PM_2.5_ and PM_10_ sensors. The laboratory calibration has been carried out at the headquarters of the CNR-IBIMET institute in Florence (Italy) against a TSI DustTrak reference instrument. Field validation has been performed across a full “heating season” (1 November 2016 to 15 April 2017), when an extensive use of domestic heating, along with unfavourable weather conditions, leads to increased pollution levels. AIRQino has been co-located at a road site in Florence where an official fixed air quality station—employing a gravimetric sampling method—was operated by the Tuscany Region Environmental Protection Agency (ARPAT). The differences marked by AIRQino with respect to most of similar existing low-cost sensor platforms include, for example, finer scores achieved in both calibration and validation processes, as well as system’s robustness, as it was deployed through a five and a half month outdoor campaign without registering sensor failures or drifts. The novelty brought by this work mainly includes: (i) a remarkable accuracy of the mere factory-calibrated PM_2.5_ and PM_10_ sensors integrated in the system; (ii) fine and reliable results achieved during both calibration and validation processes; (iii) development of a calibration procedure implementing advanced mathematical techniques to detect possible complex non-linear relationships between sensor signals and reference data.

## 2. System Development

### 2.1. System Hardware

**Figure 1:**
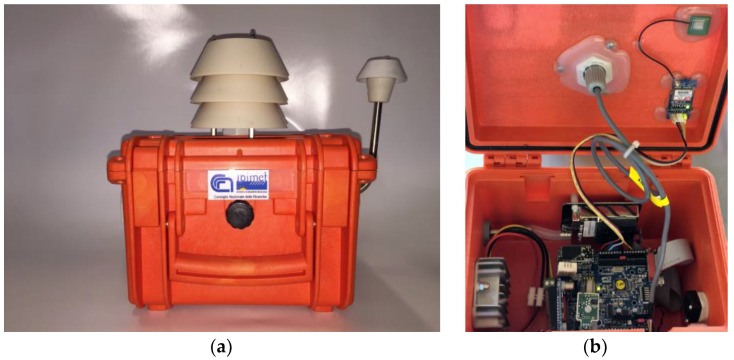
Pictures of the AIRQino monitoring station: (**a**) closed; (**b**) open.

**Figure 2:**
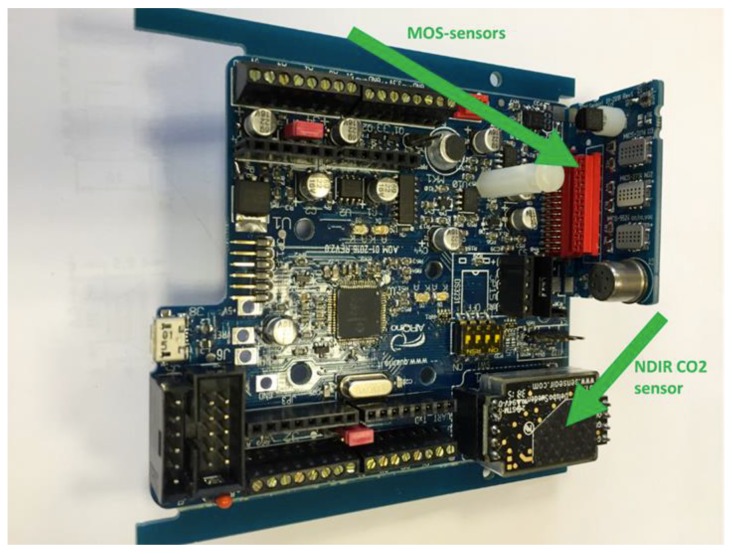
Picture of the integrated circuit board.

AIRQino (Figure 1) is a complete air quality sensors board equipped with a set of industrial integrated sensors [31]. The board is Arduino Shield compatible, integrated with low-cost and high-resolution sensors, designed to monitoring environmental parameters and atmospheric pollutants, namely: air temperature, relative humidity, CO, CO_2_, NO_2_, O_3_, VOC, PM_2.5_, PM_10_ (Figure 2). The air sampling system has been designed to meet two basic requirements: low-cost, and minimal interference with reactive gases. The air flow enters through two IP 33 ventilation devices (mod. 3540631, Fibox Inc., Glen Burnie, MD, USA) and is guaranteed by a MC20080V1 brushless fan (Sunon Inc., Brea, CA, USA) with a nominal flow-rate of 2.7 m^3^/h. The sensors, installed near the air inlet, are exposed to a high air flow-rate that guarantees a low contamination of the reactive gases (NO_2_ and O_3_). The particulate matter is aspirated through a stainless steel sampling line (8 mm internal diameter pipe) designed and tested to minimize the dimensional size of the sampled particles.

The board integrates a microprocessor unit that acquires all signals from the sensors and analyses them. Through the General Packet Radio Service (GPRS) technology, geolocated data collected from the sensors are transmitted to a webserver connected to the applications, allowing visualization of real-time observations on a web browser.

A spatial data infrastructure has been implemented, which is composed of a central Geo Database for data storage and management, a GIS engine, and a web interface. Through common web or mobile browsers, all collected data are real-time visualized in table or chart format, or tracks and spot values on a Google mashup [31].

The application scenario of the system is for continuous operation for at least 1 year without maintenance. Replacement of the sensors board—removable from the AIRQino board—is scheduled every 2 years. The system is equipped with a watchdog module for automatic reset of the CPU at scheduled intervals.

The AIRQino board can work as an Arduino shield that can access data through the PD2 (TX) and PD3 (RX) ports and provide the 5 V power supply. It can also be used in stand-alone mode using a USB interface able to data communication and 5 V power supply. The average power consumption of the board, with all sensors mounted, is about 230 mA@5 V, with peaks of about 450 mA. In stand-by mode (5 V sensors not powered), the absorption is about 65 mA@5 V. The PM sensor requires an additional average 90 mA, with peaks of 1.5 A. With special interface boards, the board can also be connected in Bluetooth or Wi-Fi modes.

The system is provided with an internal DC-DC converter unit that accepts a wide range of voltage input, from 10 V to 30 V DC. Power consumption is 200 mA@12 V DC, about 2.5 W. The system is relatively small in size (28 cm × 24 cm × 20 cm) and light in weight (1.5 kg), with a total cost of €1000.

Power management has been carefully studied and designed to obtain the best system’s performance and reliability [32]. One of power system main features is the wide range of input voltages accepted. The system is equipped with a low-cost DC-DC unit, the CPT-C5, a power converter module output 5 V, input 12/24 V, low heat, and adopts a synchronous rectification technology (i.e., 5 A long-term without any additional cooling measures). The system—particularly the AIRQino sensor board—is designed to minimize power consumption and is equipped with a CPU-controlled digital switch which turns off the sensors as well as the air intake fan when not in use [33,34,35]. During current experimental application, the system was connected to the mains without any particular power limitations, which therefore kept all sensors always on. The 2.5 W power requirement is the maximum power consumption during sensors reading and data transmission. The average power consumption depends on the sampling frequency of sensors reading and data transmission to the web server.

Analogue sensors are interrogated every second and analyzed by the CPU applying a moving average of 120 values in order to minimize oscillations and stabilize the reading. The sensors board is also equipped with a built in 5 V voltage reference to provide stable and accurate reference to the 10 bit ADC converter. A stable power supply is guaranteed by the CPT-C5 DC-DC module.

### 2.2. Sensors’ Specifications

The AIRQino station is composed of environmental, gaseous, and particulate matter sensors, whose characteristics are detailed below.

- air temperature and relative humidity: AM2315 (Adafruit, New York City, NY, USA);
- CO: TGS-2600 (Figaro Inc., Arlington Heights, IL, USA);
- CO_2_: S8 (SenseAir, Delsbo, Sweden);
- NO_2_: MiCS-2714 (SGX-Sensortech, Neuchatel, Switzerland);
- O_3_: MiCS-2614 (SGX-Sensortech);
- VOC: MiCS-5524 (SGX-Sensortech).

Particulate matter sensors for PM_2.5_ and PM_10_ (µg/m^3^), which are the focus of this study, are based on the Novasense SDS011 detector (Inovafitness, Jinan, China). This device is based on the laser scattering principle. Light scattering can be induced when particles go through the detecting area: the scattered light is transformed into electrical signals and these signals are amplified and processed. The number and diameter of particles can be obtained by analysis because the signal waveform has certain relations with the particles diameter. For these sensors it is suggested a replacement every 2–3 years depending on local environmental conditions, particularly the internal contamination caused by high level of dust in the atmosphere.

## 3. Laboratory Calibration

### 3.1. Laboratory Calibration Setup

A calibration laboratory was setup at the headquarters of CNR-IBIMET in Florence (43°47′52″ N, 11°11′31″ E), where high quality analytical devices have been operated as reference instruments: for PM_2.5_ and PM_10_ sensors calibration, the DustTrak DRX model 8533 (TSI Inc., Shoreview, MN, USA) has been used. AIRQino stations have been located outside the institute in a dedicated space: therein the same sampled air was simultaneously injected to the reference instruments through teflon tubes.

### 3.2. Reference Instrumentation for PM_2.5_ and PM_10_ Calibration

**Figure 3:**
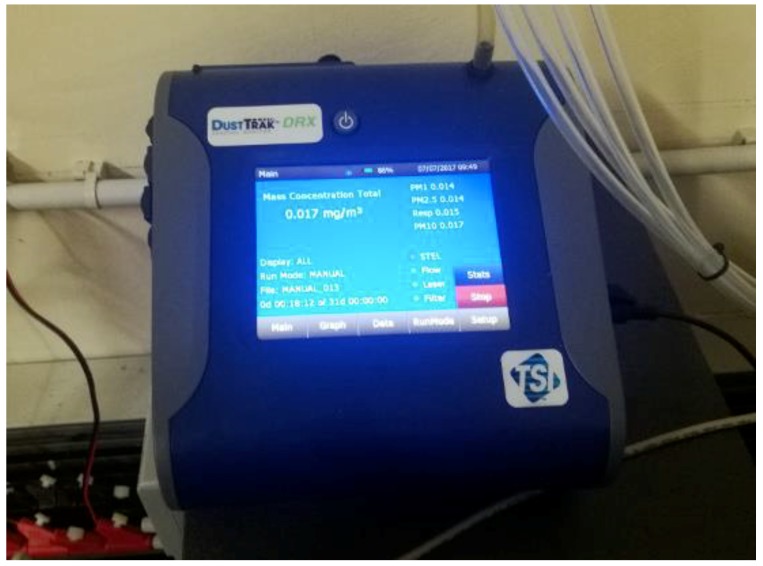
The TSI DustTrak DRX used for PM_2.5_ and PM_10_ laboratory calibration.

The TSI DustTrak desktop monitor (Figure 3) is a battery-operated, data-logging, light-scattering laser photometer that simultaneously measures size-segregated mass fraction concentrations corresponding to PM_1_, PM_2.5_, PM_4_, PM_10_ and total PM. Real-time particle mass concentration is determined by the intensity of the light scattered by the particles in the aerosol stream. The DustTrak detection range is from 0.001 to 150 mg/m^3^.

### 3.3. Calibration Procedure

**Figure 4:**
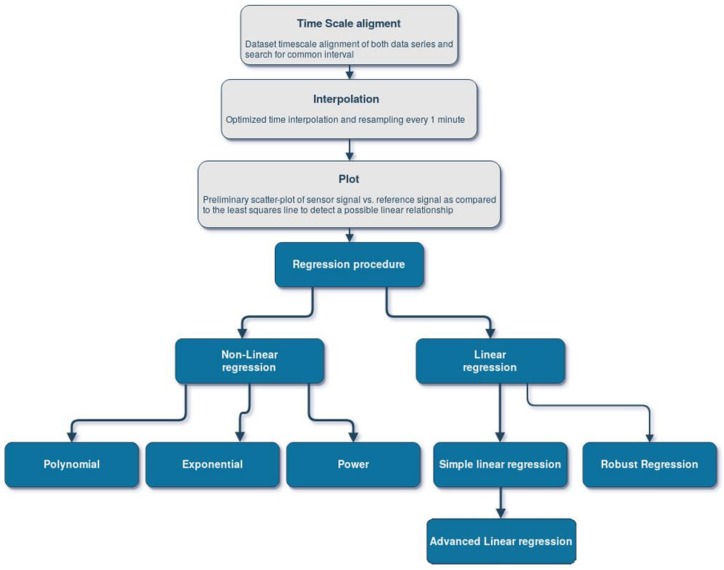
Workflow of the developed calibration procedure.

An automated procedure, developed in MATLAB and presented in Figure 4, has been implemented in order to accomplish the AIRQino laboratory calibration. PM_2.5_ and PM_10_ readings from the AIRQino and DustTrak reference stations spanned 3–15 July 2016. Data acquisition frequency was 2 min for DustTrak, while varying from 1 to 3 min for AIRQino.

Once readings have been collected from both AIRQino and DustTrak stations, the calibration procedure—as depicted in Figure 4—has been run by implementing all the following operations:

- timescale alignment of both data series along the common interval;
- optimized time interpolation and resampling every 1 min;
- preliminary scatter-plot of sensor signal vs. reference signal as compared to the least squares line to detect a possible linear relationship;
- “simple” linear regression, i.e., without outliers removal, based on least squares minimization;
- “advanced” linear regression, i.e., including analysis of residuals based on [36], and outliers removal using the Cook’s distance, a measure that combines the information of leverage and residual of the observation [37];
- robust linear regression, aimed at reducing the influence of outliers on least square fitting [38] using the M-estimation method [39], which performs an iterative weighted least squares estimation ultimately achieving a weight matrix [40]; the M-estimation function is given in the form of a weight function *w*(*e*) of residuals *e*, with the default tuning constant *k* giving coefficient estimates that are approximately 95% as statistically efficient as the ordinary least squares estimates, provided that the response has a normal distribution with no outliers [41]; in applying the M-estimation, the following weight functions have been used [42]: (i) Andrews; (ii) Bisquare; (iii) Cauchy; (iv) Fair; (v) Huber; (vi) Logistic; (vii) Talwar; (viii) Welsch;
- non-linear regressions: polynomial, i.e., (i) quadratic and (ii) cubic; (iii) exponential; (iv) power; for exponential and power regressions, a MATLAB implementation of Levenberg-Marquardt non-linear least squares algorithm has been employed which requires initial estimates of the parameters as input to successfully converge [43].

Table 1 summarizes both the linear and non-linear regression laws implemented in the calibration procedure, where *x* is the predictor (i.e., sensor signal), *F*(*x*) is the expected value (reference signal), and *β_i_* are the regression coefficients.

### 3.4. PM_2.5_ and PM_10_ Calibration Results

**Figure 5:**
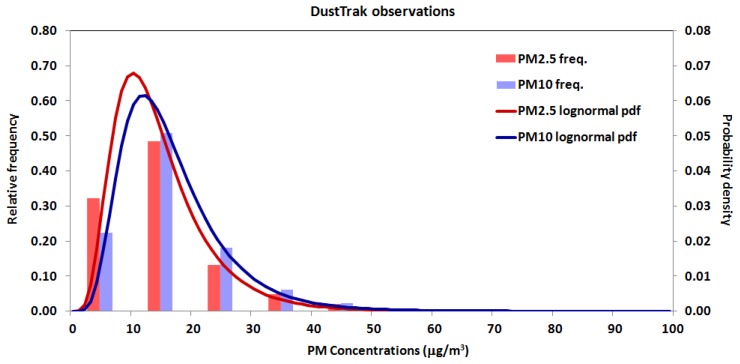
Frequency distribution and corresponding probability density function of PM_2.5_ and PM_10_ concentrations measured by the DustTrak reference instrumentation during sensors calibration.

In Figure 5 the frequency distribution of PM_2.5_ and PM_10_ concentrations measured by the DustTrak is plotted, as well as the corresponding log-normal probability density function.

**Figure 6:**
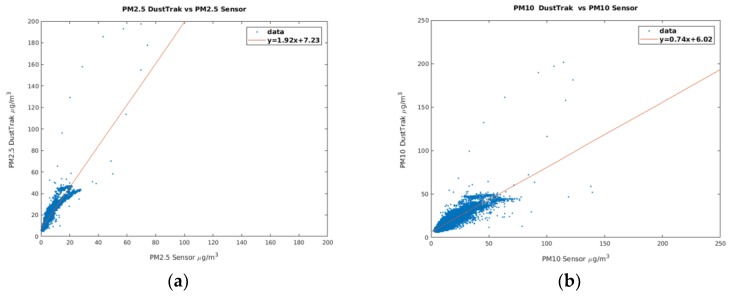
Scatter-plots of 2-min sampled sensor signals vs. reference signals observed during sensors calibration: (**a**) PM_2.5_; (**b**) PM_10_.

The PM_2.5_ and PM_10_ sensor signals compared to the reference signals observed during the calibration process are shown in Figure 6.

In Table 2 and Table 3 the statistics of the calibration procedure applied to PM_2.5_ and PM_10_ sensors are summarized, respectively, where a total of 13,222 records have been processed. To assess performances of the regression analysis, the following metrics have been used: coefficient of determination (R^2^); mean bias (MB); root mean square error (RMSE); sum of squared errors (SSE); sum of squares due to regression (SSR); total sum of squares (SST, where SST = SSE + SSR).

The SSE, measuring the total deviation of the response values from the fit to the response values, is defined as [44]:

(1) $$\overset{n}{\underset{i=1}{\sum }}{\left(y_{i}-{\hat{y}}_{i}\right)}^{2}$$

where $y_{i}$ is the *i*-th value of the variable to be predicted and ${\hat{y}}_{i}$ is the predicted value of $y_{i}$.

The SSR is defined as the sum of squared deviations of the fitted values from their mean [44]:

(2) $$\overset{n}{\underset{i=1}{\sum }}{\left({\hat{y}}_{i}-\bar{y}\right)}^{2}$$

where $\bar{y}$ is the mean value of the response variable.

The coefficient of determination R^2^ is the proportion of the total sum of squares explained by the model [44]:

(3) $$R^{2}=\mathrm{SSR}/\mathrm{SST}$$

For non-linear models as exponential and power laws, R^2^ is not a useful metric, as SST ≠ SSE + SSR in many cases, and thus R^2^ has been withdrawn from the analysis. Conversely, R^2^ is valid for linear models that use polynomials to model curvature in the data [45].

In the PM_2.5_ calibration (Table 2), the “simple” linear regression analysis returned R^2^ = 0.8095, RMSE = 3.3 µg/m^3^, and SST = 0.7743 (mg/m^3^)^2^. During the “advanced” linear regression analysis, a residual analysis was additionally performed, which highlighted the presence of outliers. To improve the regression, a cut-off threshold computed by the Cook’s distance [46] was applied, which conversely resulted in SSR value lower than in the case of “simple” linear regression: since SSR quantifies how much SST the model explains, the model was not improved by the outliers removal from the original data (3.36%) accomplished by the Cook’s distance method, which is thus not suited for this dataset. Therefore, the “advanced” linear regression resulted in better RMSE and SST values, while in a worse value for R^2^. On the contrary, with respect to the initial linear regression, the use of a robust linear regression effectively reduced the influence of outliers on least square fitting with respect to the Cook’s cut-off method, as it brought about a contemporary improvement of both SST and R^2^ values, suggesting that outliers detection is in any case a winning strategy. Very slight differences were achieved in all statistical scores from all applied robust linear regression models: in any case, Talwar proves to be the best M-estimation weight function, resulting in the overall highest value of R^2^ (0.8634) and lowest value of RMSE (2.6 µg/m^3^). The Talwar M-estimation weight function is given as:

(4) $$w\left(e\right)=\{\begin{array}{c} 1\;for\;\left|e\right|\leq k\\ 0\;for\;\left|e\right|>k \end{array}$$

with default tuning constant *k* = 2.795.

The use of polynomial regression models was in principle expected to improve the regression analysis, as a quadratic model can explain curvature in the data, while a cubic model can describe a peak-and-valley pattern in the data. Conversely, neither polynomial model proved to significantly improve the initial linear regression, as basically exhibiting the same statistical scores. Noteworthy, neither exponential and power regression models deliver a significant improvement with respect to the initial linear regression: although SSR values are largely lower than all other models (0.1531–0.3729 (mg/m^3^)^2^), RMSE and SSE values are higher, vice versa.

In general, PM_10_ calibration returned worse scores than PM_2.5_ calibration (Table 3). The initial (without outliers removal) linear regression analysis returned R^2^ = 0.6747, RMSE = 4.5 µg/m^3^, and SST = 0.8199 (mg/m^3^)^2^. Unlike the case of PM_2.5_, the “advanced” linear regression analysis applied to PM_10_ brought about an improvement in all statistical scores: R^2^ = 0.7221, RMSE = 3.2 µg/m^3^, and SST = 0.4830 (mg/m^3^)^2^. Also for PM_10_ the presence of outliers was ascertained by the residual analysis: in this case, though, application of the Cook’s cut-off method for outliers removal from the original data (3.72%) improved all the statistics. However, similarly to the case of PM_2.5_, the robust linear regression led to a further improvement: all robust linear regression models returned the highest R^2^ values, particularly if using the Talwar M-estimator (R^2^ = 0.7679), which also returned RMSE = 3.3 µg/m^3^ and SST = 0.6408 (mg/m^3^)^2^. Noticeably, neither polynomial model improved the scores achieved by the “advanced” linear model, marking only a slight improvement even against the “simple” linear model. Exponential and power models, although again showing the lowest SSR values (0.2721–0.3098(mg/m^3^)^2^), returned the highest RMSE values (4.6–4.9 µg/m^3^), thus confirming not to be the best regression solution.

The best regression models for each pollutant, along with the corresponding correlation coefficients *β*_0_ and *β*_1_ achieved during the calibration process, are summarized in Table 4: for both sensors, the robust linear regression using the Talwar M-estimator proved to be the best model.

## 4. Field Validation

**Figure 7:**
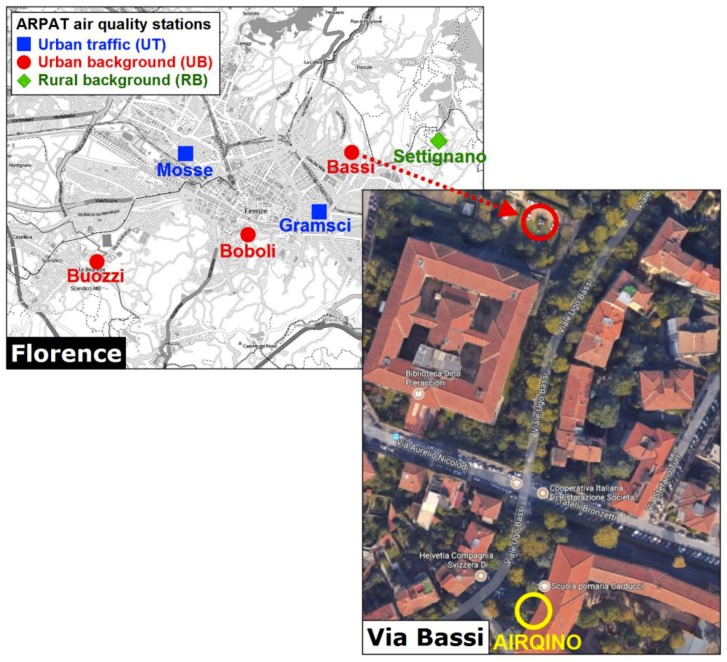
Map of air quality monitoring network operated by ARPAT in the city of Florence, and aerial view of via Bassi where the AIRQino PM_2.5_ and PM_10_ sensors were compared against the ARPAT station (Cartography sources: Bing and Google Maps).

AIRQino-integrated PM_2.5_ and PM_10_ sensors have been validated on-site against an official fixed air quality station (43°47′08″ N, 11°17′12″ E) currently in operation (i.e., an urban background station managed by ARPAT) located along via Bassi, a residential area in the city of Florence (Figure 7). ARPAT station employed a dual-channel filter-based gravimetric sampling method [47]. The AIRQino station was installed on a windowsill, about 3 m from ground level, of the Carducci School Institute, located along via Bassi as well (43°47′01″ N, 11°17′11″ E), about 200 m away from the ARPAT station.

Since daily average values of PM_2.5_ and PM_10_ concentrations are measured by the ARPAT station, field validation was limited to daily concentrations. To enhance robustness of the validation process, a monitoring campaign including the cold months was selected, i.e.,—consistently with Zikova et al. [27]—a “heating season”, when the household heaters were on and, thus, residential wood combustion contributed as an additional emission source to the PM concentrations. Based on heating regulations in Florence, this period spanned from 1 November 2016 to 15 April 2017.

**Figure 8:**
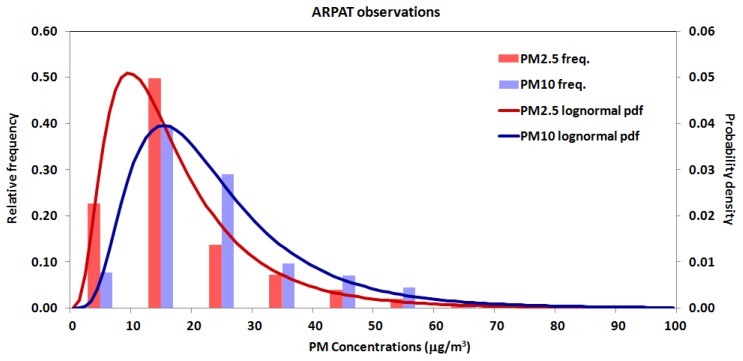
Frequency distribution and corresponding probability density function of PM_2.5_ and PM_10_ concentrations measured by the ARPAT reference station during field validation.

In comparing daily averaged concentrations measured by the AIRQino station against the ARPAT station, two different options have been considered, aimed at assessing two opposite (i.e., worst-case vs. best-case) conditions: (i) uncalibrated sensors, i.e., the mere factory-calibrated sensors, which directly return concentrations in µg/m^3^ (see Section 2.2); (ii) sensors calibrated in laboratory, i.e., those which underwent the robust linear regression using the Talwar M-estimator (see Section 3.4). Figure 8 shows the frequency distribution and related log-normal probability density function of PM_2.5_ and PM_10_ concentrations measured by the ARPAT station.

Table 5 summarizes the basic statistics of 24-h averaged PM_2.5_ and PM_10_ concentrations measured in via Bassi by the two different AIRQino configurations (i.e., with uncalibrated and calibrated sensors) as compared to the ARPAT observations.

The statistical scores of this comparison are reported in Table 6, where the following metrics have been used: mean bias (MB) and normalized mean bias (NMB); mean absolute error (MAE); root mean squared error (RMSE) and normalized root mean squared error (NRMSE); coefficient of determination (R^2^); fraction of predictions within a factor of two of observations (FAC2).

First of all, it should be stressed that uncalibrated sensors deliver quite accurate measurements, particularly in capturing day-by-day concentrations variation, as R^2^ values of 0.840 (PM_10_) and 0.900 (PM_2.5_) are achieved (Table 6). While PM_2.5_ concentrations are biased by 4.394 µg/m^3^, PM_10_ are only by 0.720 µg/m^3^, corresponding to normalized values of 21.401% and 2.863%, respectively. All ARPAT observations are over-estimated by uncalibrated AIRQino sensors.

Laboratory calibration resulted in a general improvement in the measurement of PM concentrations: this improvement was moderate in the R^2^ values (from 0.900 to 0.957 for PM_2.5_, and from 0.840 to 0.909 for PM_10_), and in the MB values for PM_10_ (from 0.720 to 0.598 µg/m^3^), while remarkable in the MB values for PM_2.5_ (from 4.394 to 0.036 µg/m^3^). Summarizing, AIRQino calibrated measurements were on average biased by less than 0.2% for PM_2.5_, and less than 2.4% for PM_10_. Also AIRQino calibrated sensors over-estimated ARPAT reference observations.

The statistical analysis has been graphically complemented by the scatter-plots between AIRQino and ARPAT measured concentrations.

**Figure 9:**
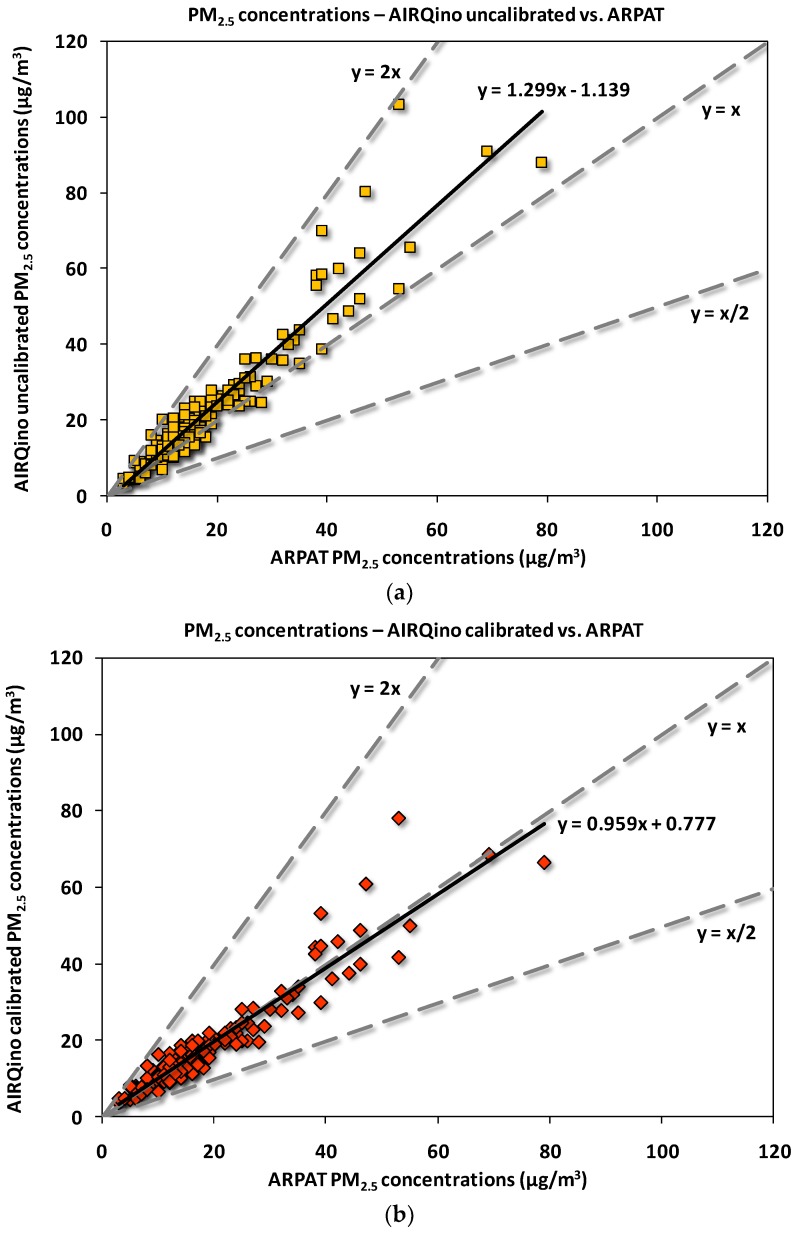
Scatter-plot of 24-h averaged PM_2.5_ concentrations (µg/m^3^) measured in via Bassi (Florence) by AIRQino (**a**) uncalibrated and (**b**) calibrated stations vs. ARPAT reference station (1 November 2016 to 15 April 2017). Dashed lines indicate the perfect agreement between the series (y = x), as well as over-estimation (y = 2x) and under-estimation (y = x/2) by a factor of two.

As for PM_2.5_ concentrations (Figure 9), the AIRQino uncalibrated station returns a clear over-estimation of ARPAT observations (Figure 9a), which is however dramatically adjusted by the calibration process (Figure 9b) so as to make the least squares line to almost match the best-fitting line (y = x). The number of points outside the dashed lines—encompassing under-estimations (y = x/2) or over-estimations (y = 2x) by a factor of two—is 2 for uncalibrated, and 0 for calibrated station: this is the same information provided by FAC2 (Table 6), indicating that in 98.7% cases estimations are within a factor of two for uncalibrated station, and in 100% they are for calibrated station.

**Figure 10:**
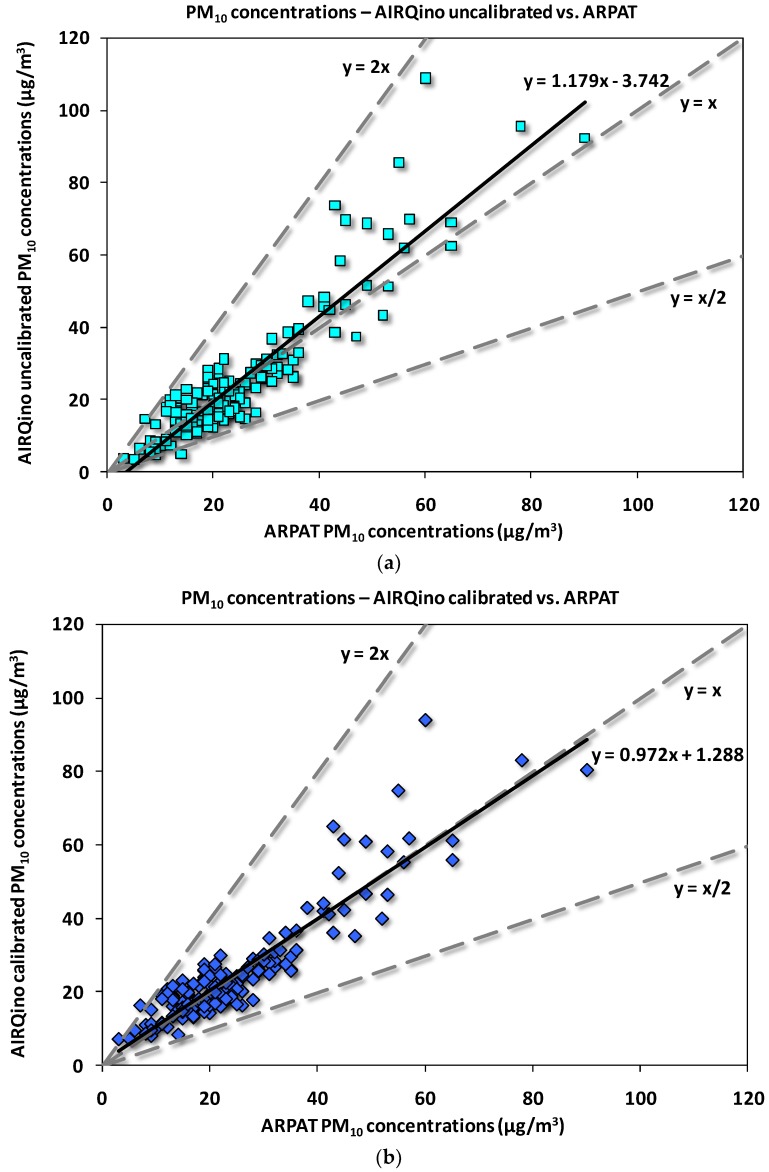
Same as Figure 9, but for PM_10_.

The scatter-plot of PM_10_ concentrations (Figure 10) generally exhibits a wider spread around the least squares line than PM_2.5_ concentrations (Figure 9), particularly for the lower concentrations. As compared to the corresponding PM_2.5_ scatter-plot, for PM_10_ a lower slope is exhibited by the trendline of uncalibrated station (Figure 10a), which is, again, basically straightened to 1:1 by the calibration process (Figure 10b). For both uncalibrated and calibrated stations, only in 2 days ARPAT PM_10_ daily concentrations are exceeded by a factor of two, i.e., FAC2 = 98.7% (Table 6).

**Figure 11:**
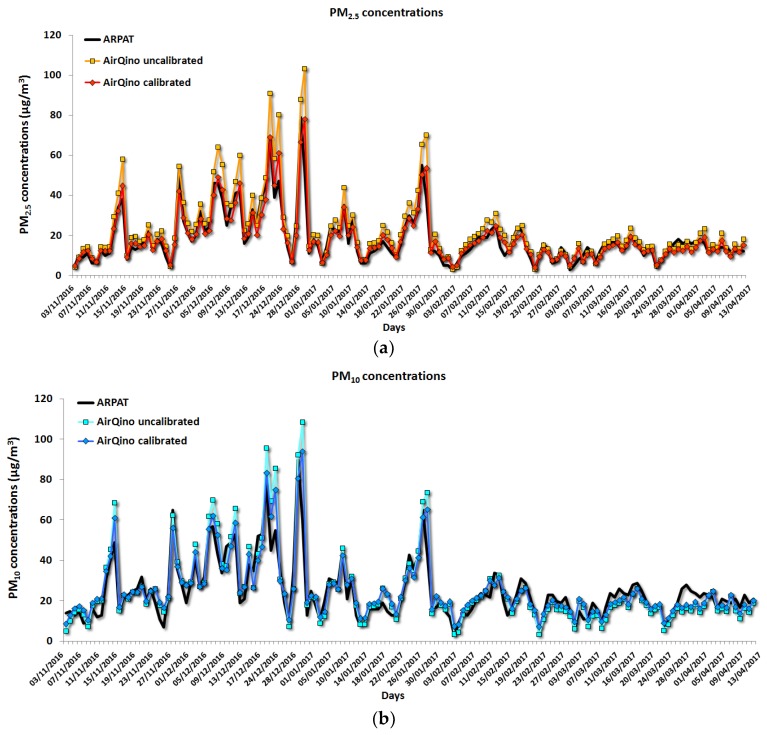
Comparison between 24-h averaged (**a**) PM_2.5_ and (**b**) PM_10_ concentrations (µg/m^3^) measured in via Bassi (Florence) by calibrated and uncalibrated AIRQino stations vs. ARPAT reference station (1 November 2016 to 15 April 2017).

Figure 11 shows the time series of uncalibrated and calibrated observations of AIRQino station as compared to the ARPAT station observations.

The AIRQino uncalibrated station is capable of well capturing the PM_2.5_ day-by-day variations (Figure 11a), as expected since R^2^ = 0.900 (Table 6). However, the accuracy improvement achieved by the calibration process is evident if analysing the pattern of the calibrated station, as it almost perfectly matches both peak and valley PM_2.5_ concentrations.

A similar general pattern is exhibited by the AIRQino station in the measurement of PM_10_ concentrations (Figure 11b), where the quite fine observations of the uncalibrated station are further improved by the calibration process, particularly in best fitting the PM_10_ peak concentrations.

## 5. Discussion

The results achieved from current laboratory calibration have been compared to other calibrations accomplished in the literature.

For example, Holstius et al. [23] carried out two field calibrations of a low-cost PM_2.5_–measuring sensor at a regulatory monitoring site in West Oakland (CA, USA) using both linear and non-linear regression models. During the first calibration, using 1-h data collected from 15 to 23 April 2013, they achieved R^2^ = 0.64–0.70 and RMSE = 4.6–5.1 µg/m^3^ against a TSI DustTrak 8530 reference station, and R^2^ = 0.55–0.60 and RMSE = 3.4–3.6 µg/m^3^ against a *β*-attenuation monitor (BAM) reference station. During the second calibration, using 24-h data collected from 1 Aug to 15 Nov 2013, they achieved R^2^ = 0.72 against a BAM reference station. Compared to both these PM_2.5_ calibrations, scores from present calibration are finer, as R^2^ = 0.8634 and RMSE = 2.6 µg/m^3^ (Table 2).

Another field calibration was performed by Velasco et al. [1] in the city of Turin (Italy) after co-locating their low-cost sensor to an ARPA Piedmont fixed station using 24-h PM_10_ concentrations collected during the autumn 2015: by employing a linear regression, they achieved a Pearson correlation coefficient (*r*) of 0.61, whose square value is lower than R^2^ = 0.7679 obtained from present PM_10_ laboratory calibration (Table 3).

On the contrary, scores from current PM_2.5_ calibration are outperformed by those obtained by Shao et al. [24], who compared their low-cost PM_2.5_ sensing system against a Tapered Element Oscillating Microbalance (TEOM) reference station in Wolongqiao (China) based on 1-h data collected from 7 to 14 March 2016. In doing so, they used a non-linear model as a function of PM readings and scattered light fluxes. Their sample were best fitted by using a quadratic polynomial regression, which returned R^2^ values between 0.901 and 0.945 (vs. R^2^ = 0.8634 from present calibration).

In any case, the far larger sample size (N = 13,222) used for Florence laboratory calibration with respect to those cited above is worth noticing, thus showing reliability and stability of calibration results. Furthermore, since only employing PM sensors readings as a regressor, simplicity of the currently-implemented PM calibration model—and thus its easier portability to other contexts/applications—should also be highlighted. This marks a clear distinction from multi-linear regressions or non-linear multi-variate models such as those that also consider relative humidity (e.g., [16,48]), air temperature (e.g., [48]), or scattered light fluxes (e.g., [24]) as regressors.

The calibration procedure originally developed and applied here (Figure 4) represents an added value, particularly as implementing several advanced mathematical and statistical techniques capable of exploring and detecting possible complex non-linear relationships between sensor signals and reference data, such us plots of residuals, which are very powerful methods to check the validity of linear relationship assumptions and provide information on how to improve the model (i.e., robust regression). It could therefore establish, along with very refined tools such as those implemented, e.g., by [49,50], as a state-of-the-art methodology for low-cost sensing calibration. Although such procedure is well capable of detecting and correcting non linearities in the sensors response, the results obtained here reveal a strong linearity of these detectors across a large range of environmental conditions (the minimum length of a dataset, to capture a complete sensor output, includes a day-night cycle in order to evaluate the presence of some periodical patterns), leading to the same scores for linear and non linear fitting models (Table 3).

The scores resulting from AIRQino field validation have been also compared with validation of similar low-cost systems. For example, if considering concentrations measured at the same 24-h time scale, a straight comparison can be made with the field validation carried out by Zikova et al. [27] of their PM_2.5_-measuring system in the city of Rochester (NY, USA). Therein they placed their systems at 26–27 residential locations and compared them against a TEOM reference station across two distinct “heating periods”: (i) early December 2015 to early April 2016; (ii) end of October 2016 to early April 2017. During period (i), only in 1.1% locations an *r* coefficient higher than 0.9 (corresponding to *r*^2^ = 0.81) was achieved, while during period (ii) this percentage reduced to 0.9% locations. Therefore, temporal variability of PM_2.5_ measurements from the majority of these units is outperformed by the one exhibited by the AIRQino PM_2.5_ measurements (R^2^ = 0.900–0.957, Table 6).

If also comparing Florence daily-based validation with those (more challenging) employing hourly measured concentrations, the PM_2.5_ application by Zikova et al. [26] can be analysed. In [26], 66 sensing units were co-located with a Grimm laser particle spectrometer in the city of Potsdam, NY, and a weighted least square linear regression was applied through two outdoor monitoring campaigns: (i) using 58 units from 15 to 17 October 2015; (ii) using 9 units from 20 to 23 November 2015. During campaign (i), for 84% units they found *r* > 0.80, and for 36% *r* > 0.95; during campaign (ii), for 60% units they found *r* > 0.60, and for 16% *r* > 0.80. Overall, they also achieved RSME values of about 7 and 8 µg/m^3^ for the two campaigns, respectively. These scores, although on a 1-h rather than 24-h basis, are lower than those achieved within PM_2.5_ Florence validation.

Based on hourly values, Mukherjee et al. [25] validated two different low-cost PM sensors over a 12-week campaign (14 April to 6 July 2016) in the Cuyama Valley (CA, USA) using a Grimm and a BAM as reference instruments. In measuring PM_2.5_ concentrations, they at best achieved R^2^ = 0.71 against the Grimm; for PM_10_ concentrations, they at best achieved R^2^ = 0.84 against the Grimm, and R^2^ = 0.81 against the BAM. By comparison, for both PM_2.5_ and PM_10_ measurements Florence validation scores are finer, although they are achieved based on a smaller sample size (N = 155 vs. 825–1755).

AIRQino field validation skills were also better when compared against the different microsensor systems by various developers installed side-by-side with reference analysers and then assessed in the framework of the 1st EuNetAir Air Quality Joint Intercomparison Exercise organized in Aveiro (Portugal) from 13 to 27 October 2014 [51]. Although compared against 1-h averaged concentrations, for both PM_2.5_ and PM_10_ sensors current accuracy was largely higher based on all considered metrics (see Table 4 therein). For PM_10_ sensor, in particular, the significantly different performances may be graphically appreciated by comparing current scatter-plots (Figure 10) to those presented in Figure 5 therein.

A high resolution field validation was performed by Sun et al. [16] in measuring PM_2.5_ concentrations after deploying their system in Hong Kong from 16 to 18 Jan 2015, using a non-linear model as a function of PM readings and relative humidity. Compared against an official reference station, at 5-min resolution they found R^2^ = 0.92, NMB = 13%, and MAE = 5.5 µg/m^3^, again outperformed by currently achieved scores. On the contrary, a comparable accuracy in PM_2.5_ measurement was achieved in the above-cited field validation by Shao et al. [24]: based on a sample size similar to that of the present work (N = 158), against a TEOM reference station in Wolongqiao (China) they found R^2^ = 0.948–0.966 and RMSE = 8.375–10.408 µg/m^3^.

The sensors deployed in this study (by Novasense, see Section 2.2) came with their own factory calibration, that gave acceptable results, although generally overestimating the reference measurements by 18–30% (Figure 9 and Figure 10). Using directly the factory calibration leads sometimes to more accurate measurements than those achieved after field calibration within the above-cited literature works. For PM_10_ sensor, for example, R^2^ values of 24-h concentrations (0.84, Table 6) are higher than those (0.61) achieved in the field calibration by Velasco et al. [1], with mean concentrations also slightly biased (2.863%). For PM_2.5_ concentrations, in reproducing temporal variation the factory-calibrated sensor returned finer scores (R^2^ = 0.90, Table 6) than those achieved by Mukherjee et al. [25] (R^2^ = 0.71), Holstius et al. [23] (R^2^ = 0.72), and the majority of sensors validated by Zikova et al. [26,27]. Similarly, the MAE scores are finer than those reported by Sun et al. [16] (4.827 vs. 5.5 µg/m^3^); on the contrary, with respect to the same studies, the achieved MB and RMSE scores are worse, thus effectively requiring a proper laboratory calibration. However, present results show that the best performances are obtained after a laboratory calibration of each sensor against a light-scattering laser photometer (Table 6). It is worth noting that those performances were assessed in the field against an independent instrument employing a physically different (i.e., gravimetric) sampling method.

It is clear that, aside from the mere sensing accuracy during both the calibration and validation processes, a number of further requirements shall be met by the low-cost systems, as recommended, e.g., by the EPA’s Air Sensor Guidebook [20], including their consistency, stability and durability in real-world outdoor conditions [6]. Under this respect, robustness of the AIRQino station is worth noticing, as it was seamless deployed for a five and a half month outdoor campaign without registering significant sensor failures or drifts. Outdoor validations across comparably long periods are not particularly frequent in the literature, with few exceptions such as, e.g., the one cited above by Zikova et al. [27] of 26–27 PM_2.5_-measuring elements, deployed across two “heating seasons” as well.

## 6. Conclusions and Future Work

Key findings of the current work include: (i) a remarkable accuracy of the mere factory-calibrated PM_2.5_ and PM_10_ sensors integrated in the system, particularly in capturing concentrations temporal variation; this proved to be a fair starting point for later sensors calibration and validation; (ii) fine and reliable results achieved during sensors laboratory calibration; (iii) fine scores achieved during sensors field validation; (iv) robustness of the developed sensing system, seamless deployed through a five and a half month outdoor campaign with no appreciable sensor failure or drift.

Since field validation was performed against a dataset of 24-h averaged PM concentrations, in a future work a test of PM sensors at higher temporal resolution will be addressed. Furthermore, PM sensors will be tested against higher ambient PM concentrations.

Future work’s directions should include extension of both laboratory calibration and field validation to the gas sensors (i.e., O_3_, NO_2_, CO) already integrated in the system. Unlike the present case when 24-h averaged PM concentrations have been used, for gas sensors validation different statistical scores should be expected since 1-h averaged concentrations will be used. This task will take advantage of the calibration tool developed here, as well as new robust calibration models (e.g., [52,53]) which will be thoroughly analysed for possible use.

To further test real-world consistency, stability and durability of the AIRQino station, its deployment over longer periods (one full year) as well as more aggressive environments (e.g., coastline sites) are also envisaged in the near future.

## Figures and Tables

**Table 1:** Regression laws implemented in the calibration procedure.

Regression Laws	Equation
Linear	F(x) = *β*_0_ + *β*_1_ x
Quadratic	F(x) = *β*_0_ + *β*_1_ x + *β*_2_ x^2^
Cubic	F(x) = *β*_0_ + *β*_1_ x + *β*_2_ x^2^ + *β*_3_ x^3^
Exponential	F(x) = *β*_0_ exp(*β*_1_ x)
Power	F(x) = *β*_0_ x*^β^*^1^

**Table 2:** Statistical scores of PM_2.5_ sensor laboratory calibration (N = 13,222).

Regression Model	R^2^	MB (µg/m^3^)	RMSE (µg/m^3^)	SSR (mg/m^3^)^2^	SSE (mg/m^3^)^2^	SST (mg/m^3^)^2^
Linear						
Without outlier removal	0.8095	−0.0342	3.3	0.6228	0.1475	0.7743
With outlier removal (3.36%)	0.7655	0.0388	2.6	0.2793	0.0852	0.3645
Robust linear						
Andrew	0.8499	−0.2461	2.7	0.5855	0.1033	0.6888
Bisquare	0.8501	−0.2456	2.7	0.5856	0.1032	0.6888
Cauchy	0.8484	−0.2453	2.8	0.5841	0.1043	0.6885
Fair	0.8447	−0.2209	2.8	0.5897	0.1084	0.6981
Huber	0.8532	−0.2446	2.7	0.5857	0.1007	0.6864
Logistic	0.8478	−0.2344	2.8	0.5876	0.1054	0.6931
**Talwar**	**0.8634**	**−0.1993**	**2.6**	**0.5785**	**0.0915**	**0.6701**
Welsh	0.8499	−0.2453	2.7	0.5853	0.1033	0.6887
Non-linear						
Quadratic	0.8098	−0.0462	3.3	0.6270	0.1473	0.7743
Cubic	0.8197	−0.1001	3.2	0.6347	0.1396	0.7743
Exponential	-	−0.7755	5.4	0.3729	0.2629	0.7683
Power	-	−0.0643	3.4	0.1531	0.6513	0.7683

**Table 3:** Statistical scores of PM_10_ sensor laboratory calibration (N = 13,222).

Regression Model	R^2^	MB (µg/m^3^)	RMSE (µg/m^3^)	SSR (mg/m^3^)^2^	SSE (mg/m^3^)^2^	SST (mg/m^3^)^2^
Linear						
Without outlier removal	0.6747	0.0836	4.5	0.5532	0.2667	0.8199
With outlier removal (3.72%)	0.7221	0.0312	3.2	0.3448	0.1342	0.4830
Robust linear						
Andrew	0.7499	−0.1249	3.5	0.4998	0.1666	0.6665
Bisquare	0.7500	−0.1240	3.5	0.4999	0.1665	0.6665
Cauchy	0.7485	−0.1184	3.5	0.5000	0.1679	0.6680
Fair	0.7459	−0.0977	3.6	0.5056	0.1721	0.6778
Huber	0.7480	−0.1043	3.5	0.5023	0.1692	0.6715
Logistic	0.7481	−0.1047	3.5	0.5039	0.1696	0.6735
**Talwar**	**0.7679**	**−** **0** **.** **1109**	**3.3**	**0.4921**	**0.1486**	**0.6408**
Welsh	0.7500	−0.1204	3.5	0.5005	0.1667	0.6673
Non-linear						
Quadratic	0.6981	0.1574	4.3	0.5806	0.2475	0.8199
Cubic	0.7081	0.0115	4.3	0.5724	0.2393	0.8199
Exponential	-	−0.1506	4.9	0.3098	0.3892	0.8145
Power	-	−0.0556	4.6	0.2721	0.6122	0.8145

**Table 4:** Statistical summary of the best regression models achieved from PM_2.5_ and PM_10_ sensors laboratory calibration (N = 13,222).

Pollutant	Regression Model	*β*_0_	*β*_1_	R^2^	MB (µg/m^3^)	RMSE (µg/m^3^)	SSR (mg/m^3^)^2^	SSE (mg/m^3^)^2^	SST (mg/m^3^)^2^
PM_2.5_	Robust linear: Talwar	6.3	2.0	0.8634	−0.8634	2.6	0.5785	0.0915	0.6701
PM_10_	Robust linear: Talwar	5.0	0.7	0.7679	−0.7679	3.3	0.4921	0.1486	0.6408

**Table 5:** Statistical summary of 24-h averaged PM_2.5_ and PM_10_ concentrations (µg/m^3^) measured in via Bassi (Florence) by calibrated and uncalibrated AIRQino stations, and ARPAT reference station (1 November 2016 to 15 April 2017).

Pollutant	Station	Mean	Standard Deviation	Range
PM_2.5_	ARPAT	18.45	12.64	3.00–79.00
	AIRQino uncalibrated	22.85	17.32	3.11–103.53
	AIRQino calibrated	18.49	12.79	3.92–78.06
PM_10_	ARPAT	24.80	14.31	3.00–90.00
	AIRQino uncalibrated	25.52	18.41	3.49–108.79
	AIRQino calibrated	25.40	15.17	7.25–94.00

Data sample: 155; valid data: 96.87%. No. exceedances of PM_10_ daily limit value (50 µg/m^3^): 11.

**Table 6:** Statistical scores of the comparison in measuring 24-h averaged PM_2.5_ and PM_10_ concentrations (µg/m^3^) between calibrated and uncalibrated AIRQino stations vs. ARPAT reference station (1 November 2016 to 15 April 2017).

Pollutant	Score	AIRQino Station	
		Uncalibrated	Calibrated
PM_2.5_	MB (µg/m^3^)	4.394	0.036
	NMB (%)	21.401	0.196
	MAE (µg/m^3^)	4.827	2.683
	RMSE (µg/m^3^)	7.955	4.056
	NRMSE (%)	38.746	21.959
	R^2^	0.900	0.957
	FAC2	0.987	1.000
PM_10_	MB (µg/m^3^)	0.720	0.598
	NMB (%)	2.863	2.384
	MAE (µg/m^3^)	5.103	4.309
	RMSE (µg/m^3^)	7.802	6.084
	NRMSE (%)	31.011	24.240
	R^2^	0.840	0.909
	FAC2	0.987	0.987

Data sample: 155; valid data: 96.87%.
